# Neuromodulatory Responses Elicited by Intermittent versus Continuous Transcranial Focused Ultrasound Stimulation of the Motor Cortex in Rats

**DOI:** 10.3390/ijms25115687

**Published:** 2024-05-23

**Authors:** Tsung-Hsun Hsieh, Po-Chun Chu, Thi Xuan Dieu Nguyen, Chi-Wei Kuo, Pi-Kai Chang, Kai-Hsiang Stanley Chen, Hao-Li Liu

**Affiliations:** 1 School of Physical Therapy, Graduate Institute of Rehabilitation Science, Chang Gung University, Taoyuan 33302, Taiwan; dieunguyenpt@gmail.com (T.X.D.N.); kiwi725@gmail.com (C.-W.K.); tyrant001dx@gmail.com (P.-K.C.); 2 Neuroscience Research Center, Chang Gung Memorial Hospital, Linkou, Taoyuan 33305, Taiwan; 3 Healthy Aging Research Center, Chang Gung University, Taoyuan 33302, Taiwan; 4 Department of Electrical Engineering, National Taiwan University, Taipei 10617, Taiwan; bargsonchu@ntu.edu.tw; 5 Department of Neurology, National Taiwan University Hospital Hsinchu Branch, Hsinchu 300195, Taiwan

**Keywords:** transcranial focused ultrasound, neuromodulation, motor-evoked potentials, plasticity, rats

## Abstract

Transcranial focused ultrasound stimulation (tFUS) has emerged as a promising neuromodulation technique that delivers acoustic energy with high spatial resolution for inducing long-term potentiation (LTP)- or depression (LTD)-like plasticity. The variability in the primary effects of tFUS-induced plasticity could be due to different stimulation patterns, such as intermittent versus continuous, and is an aspect that requires further detailed exploration. In this study, we developed a platform to evaluate the neuromodulatory effects of intermittent and continuous tFUS on motor cortical plasticity before and after tFUS application. Three groups of rats were exposed to either intermittent, continuous, or sham tFUS. We analyzed the neuromodulatory effects on motor cortical excitability by examining changes in motor-evoked potentials (MEPs) elicited by transcranial magnetic stimulation (TMS). We also investigated the effects of different stimulation patterns on excitatory and inhibitory neural biomarkers, examining c-Fos and glutamic acid decarboxylase (GAD-65) expression using immunohistochemistry staining. Additionally, we evaluated the safety of tFUS by analyzing glial fibrillary acidic protein (GFAP) expression. The current results indicated that intermittent tFUS produced a facilitation effect on motor excitability, while continuous tFUS significantly inhibited motor excitability. Furthermore, neither tFUS approach caused injury to the stimulation sites in rats. Immunohistochemistry staining revealed increased c-Fos and decreased GAD-65 expression following intermittent tFUS. Conversely, continuous tFUS downregulated c-Fos and upregulated GAD-65 expression. In conclusion, our findings demonstrate that both intermittent and continuous tFUS effectively modulate cortical excitability. The neuromodulatory effects may result from the activation or deactivation of cortical neurons following tFUS intervention. These effects are considered safe and well-tolerated, highlighting the potential for using different patterns of tFUS in future clinical neuromodulatory applications.

## 1. Introduction

Transcranial focused ultrasound stimulation (tFUS) is an advanced neuromodulation technique that delivers acoustic energy with exceptional spatial resolution [1,2]. Unlike conventional neuromodulation approaches that employ electrical or magnetic fields, tFUS uses acoustic energy to reach deep brain structures such as the thalamus, basal ganglia, cerebellum, and cortical areas through the skull [3,4]. The underlying principle of tFUS involves neuromodulating mechanically sensitive ion channels through the application of low-intensity, focused ultrasound waves [5,6]. This neuromodulation can lead to temporary changes in neuronal excitability, enabling researchers and clinicians to either stimulate or suppress neural activity in targeted brain regions. The versatility of tFUS in adjusting its parameters, such as frequency, intensity, and focal area size, allows for tailored treatments that can be optimized for individual patients and specific neurological conditions. However, tFUS faces challenges and limitations. Determining the optimal ultrasound parameters (frequency, intensity, stimulation pattern, and focal area) for effective neuromodulation remains crucial for enhancing stimulation effectiveness. Additionally, accurately focusing on the desired brain region with high precision, while also minimizing potential adverse effects on the surrounding tissues, presents a considerable challenge.

Furthermore, research indicates that the pattern of stimulation, such as intermittent versus continuous, can have different neuromodulatory effects and impact brain excitability in distinct ways [7]. For example, in repetitive transcranial magnetic stimulation (rTMS) protocols, intermittent theta burst stimulation (iTBS), with its pattern of 2 s of stimulation followed by an 8 s pause, can enhance cortical excitability, while continuous theta burst stimulation (cTBS), which applies continuous stimulation, tends to inhibit cortical excitability [8]. This distinction underscores the complexity and potential of brain stimulation techniques in neuromodulation.

As advancements in tFUS technology progress, ongoing research aims to further refine its parameters and enhance its efficacy. A major question that has not been fully resolved is how to select the exact ultrasound parameters to modulate neural activity (either excitatory or inhibitory). Recent studies have applied transcranial magnetic stimulation (TMS) to evaluate the changes of motor-evoked potentials (MEPs) for identifying the neuromodulation effects of tFUS on the motor cortex [9,10,11]. In our earlier study, we have also established a rat model to assess motor cortical excitability evaluated by TMS following tFUS [9]. This rat TMS–tFUS model may serve as a valuable platform for bridging animal studies and human applications. It was observed that low-intensity tFUS could induce a prolonged inhibitory response in motor excitability without causing tissue damage, as confirmed by histological analysis [9].

However, the optimal protocols for facilitating or inhibiting effects through tFUS have yet to be determined. This study aims to investigate the effects of two different tFUS protocols (intermittent and continuous) on motor cortical excitability. It is hypothesized that intermittent tFUS would facilitate, whereas continuous tFUS would inhibit, cortical excitability. Additionally, to identify the underlying mechanisms and the safety of such protocols, we further assess the excitatory and inhibitory neural biomarkers and glial fibrillary acidic protein (GFAP) expression through immunohistochemistry staining following acoustic exposure.

## 2. Results

The neuromodulatory effects of tFUS were assessed by observing changes in the motor evoked-potential (MEP) elicited by TMS in the motor cortex of the rat brain. Furthermore, to address safety concerns associated with tFUS and to verify the changes in neuronal activity induced by different patterned tFUS protocols (intermittent tFUS or continuous tFUS), immunohistochemistry (IHC) analyses of GFAP staining and the expression levels of c-Fos and GAD-65 were conducted.

### 2.1. Effects of Intermittent and Continuous tFUS on Motor Cortical Excitability

**Figure 1:**
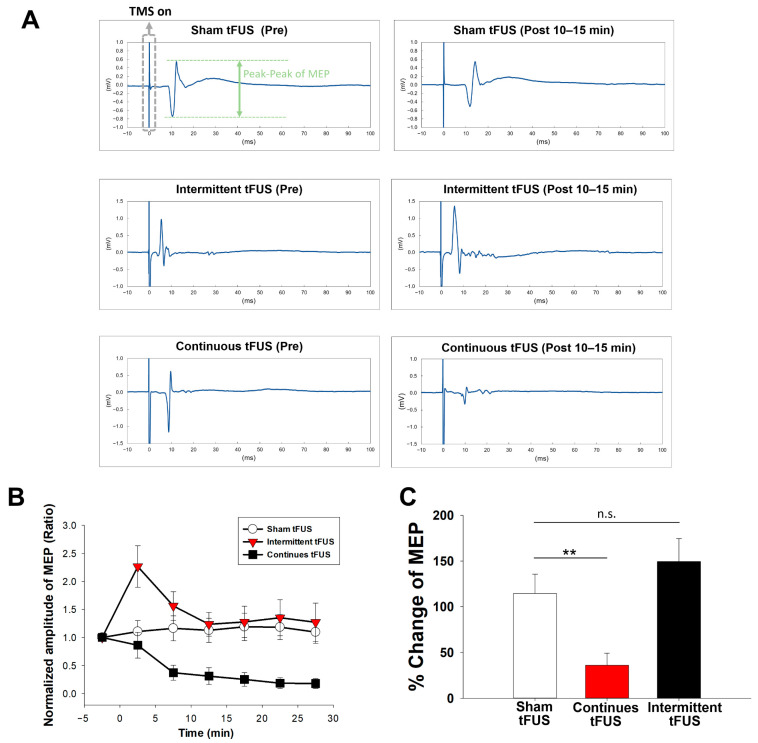
Representative motor-evoked potential (MEP) responses of a rat before and after receiving either sham, intermittent, or continuous transcranial focused ultrasound stimulation (tFUS) are displayed for each measurement period (**A**). The average normalized MEP amplitudes across the three intervention protocols (sham, intermittent, or continuous tFUS) are shown (**B**). The mean cortical excitability responses were calculated for each intervention group within 30 min following the different stimulations (**C**). Data are presented as mean ± SEM for 7–8 rats per group. ** indicates *p* < 0.01, denoting significant differences in MEP size post-intervention compared with the baseline measured before tFUS, as determined by a post-hoc Fisher’s LSD test. n.s.: no significance.

To evaluate the neuromodulatory effects of tFUS, animals were anesthetized and then positioned in a stereotaxic apparatus. A Sonopore ultrasound exposure transducer with a fundamental frequency of 1 MHz was utilized. The MEPs were recorded for 5 min before tFUS to serve as the baseline. Then, the recording of MEPs continued for 30 min to observe the post-tFUS effect. Figure 1A illustrates the typical MEP changes observed under continuous or intermittent tFUS application.

Continuous tFUS application resulted in the inhibition of MEP amplitudes at all observed time points, whereas intermittent tFUS facilitated MEPs. No significant changes were found in the MEPs for each time point after sham treatment (*p* > 0.05) (Figure 1B). A two-way repeated-measures ANOVA revealed significant main effects of time (F_(2.69, 51.16)_ = 5.702, *p* = 0.003) and group over time on MEPs (F_(2, 19)_ = 7.69, *p* = 0.004), with a significant interaction between time and group (F_(5.385, 51.158)_ = 4.44, *p* = 0.002). Post-hoc tests confirmed that continuous tFUS applied to the motor cortex significantly suppressed MEP amplitudes compared to the sham group at all post-intervention time points (all *p* < 0.05). Intermittent tFUS showed significant facilitation only in the first time interval (0–5 min post-stimulation) when compared with sham stimulation (*p* = 0.02).

To assess the neuromodulatory effects across different stimulation groups, these percentage changes were further analyzed. The percentage change in MEPs was determined by comparing the average MEP amplitude at each testing time point (i.e., 0–5, 5–10, 10–15, 15–20, 20–25, and 25–30 min) after sham, continuous, or intermittent tFUS. Figure 1C illustrates the average MEP levels across 30 min following sham, continuous, or intermittent tFUS. The responses to continuous tFUS showed a significant reduction compared to sham stimulation (*p* = 0.008), whereas no significant difference was observed between sham and intermittent tFUS (*p* = 0.31).

### 2.2. Histological Assays

**Figure 2:**
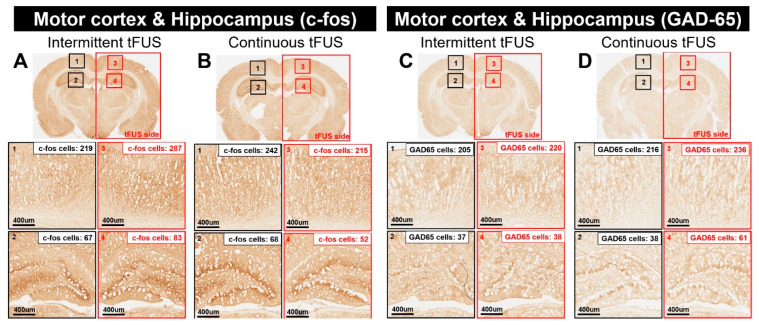
Representative immunohistochemically stained sections highlighting the regions of interest (ROI) comprising the motor cortex and hippocampus. The data illustrate the quantity of c-Fos-positive cells within the ROI following intermittent transcranial focused ultrasound stimulation (tFUS) (**A**) and continuous tFUS (**B**). Similarly, the number of GAD-65-positive cells in the motor cortex and hippocampus is shown after exposure to intermittent tFUS (**C**) and continuous tFUS (**D**).

To determine whether intermittent or continuous tFUS activates neurons and GABAergic synaptic terminals, we performed experiments on rats, examining the expression of the immediate early genes c-Fos and GAD-65 following either type of tFUS intervention. Following 20 min of tFUS intervention, the rats were transcardially perfused and their brains were analyzed through IHC to assess the expression levels of c-Fos and GAD-65. These indicators of neuronal and synaptic activity were used to confirm the changes in neuronal activity elicited by both continuous and intermittent tFUS (Figure 2).

The current results indicate that intermittent tFUS led to an increase in the number of c-Fos-positive cells within the stimulated regions, including the motor cortex and hippocampus, when compared to the unstimulated hemisphere (Figure 2A). Conversely, continuous tFUS resulted in a reduced expression of c-Fos in the motor cortex and hippocampus when compared to the unstimulated side (Figure 2B).

According to our GAD-65 analysis, rats subjected to intermittent tFUS did not show an obvious increase in GAD-65 expression (Figure 2C). However, continuous tFUS was associated with an increase in GAD-65 expression within the stimulated areas (motor cortex and hippocampus) (Figure 2D). These findings demonstrate that intermittent and continuous tFUS specifically activate excitatory and inhibitory neurons, respectively.

**Figure 3:**
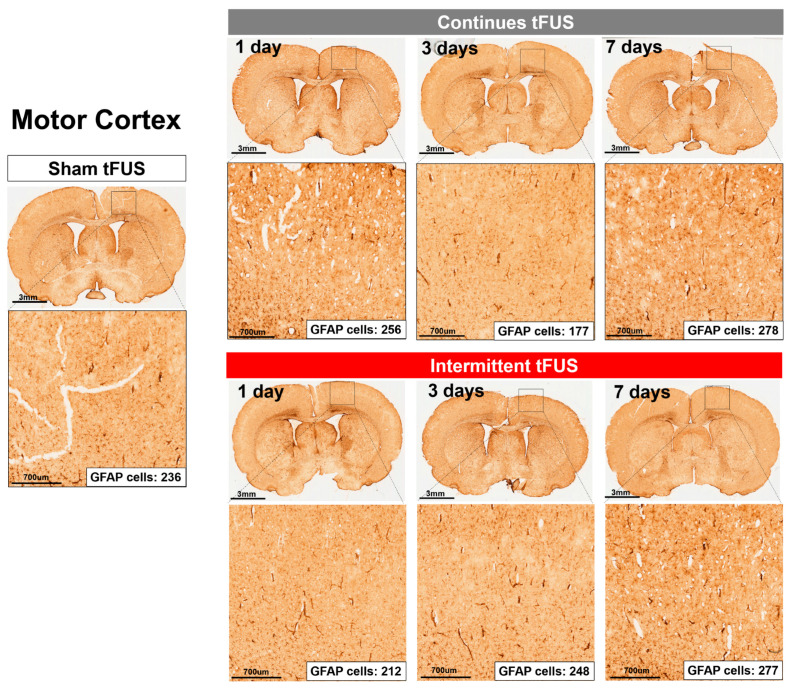
Representative images of glial fibrillary acidic protein (GFAP) immunostaining in the motor cortex of rats sacrificed 1 day, 3 days, and 7 days after receiving sham, continuous, or intermittent tFUS are presented. Compared to rats that received sham stimulation, no obvious astrogliosis was observed at or near the sonicated sites in the brains of those treated with either continuous or intermittent tFUS.

**Figure 4:**
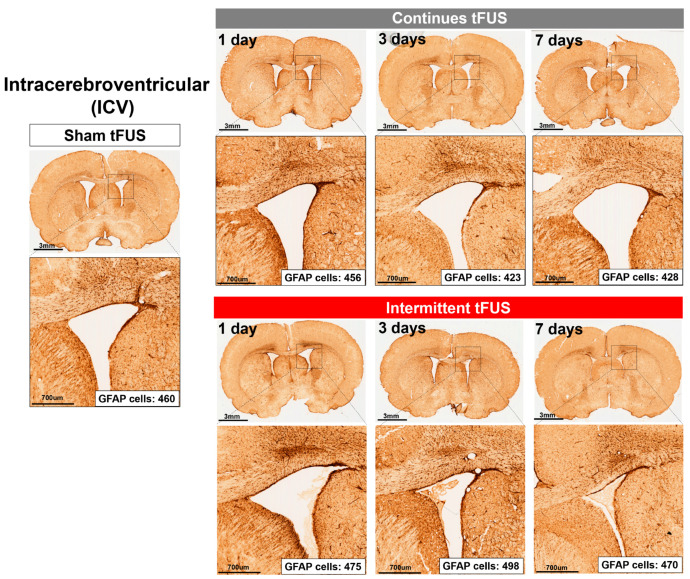
Representative images showcasing immunostaining for glial fibrillary acidic protein (GFAP) within the intracerebroventricular (ICV) area of rats sacrificed at 1 day, 3 days, and 7 days post-exposure to either sham, continuous, or intermittent tFUS intervention. In comparison to the sham group, no obvious astrogliosis was detected at or around the sonication locations in brains subjected to either continuous or intermittent tFUS treatment.

We then assessed the safety of tFUS by examining the expression of GFAP-immunoreactive astrocytes, which are known to activate in response to brain injury [12,13]. IHC staining was used to detect GFAP-positive cells 1 day, 3 days, and 7 days after stimulation. Our IHC analyses revealed no obvious increase in GFAP-positive cells in the motor cortex (Figure 3) or the intracerebroventricular (ICV) area (Figure 4) of a rat subjected to continuous or intermittent tFUS, compared to a rat receiving sham stimulation.

## 3. Discussion

This study confirmed that tFUS, using different protocol patterns, significantly influences cortical excitability through distinct neuromodulatory effects. Specifically, intermittent tFUS was found to immediately enhance MEP amplitudes, which then gradually returned to baseline levels. In contrast, continuous tFUS consistently reduced MEPs, with this suppression lasting up to 30 min. Beyond electrophysiological measurements using MEPs, we also conducted immunohistochemistry examinations of c-Fos and GAD-65 to verify that ultrasound-induced neuromodulatory effects, respectively, activated excitatory and inhibitory neurons. Notably, we observed no increase in GFAP expression at 1, 3, or 7 days post-tFUS, indicating no obvious brain injury from the intervention. Our findings underscore the safety and efficacy of tFUS as a neuromodulation technique capable of inducing neural plasticity and activating neural cells, leading to the exploration of the potential use of tFUS clinical applications in treating neurological and neuropsychiatric disorders.

tFUS is an emerging, non-invasive neuromodulation tool for deep brain stimulation that has shown promise in treating various neurological and psychiatric conditions [14,15,16]. Following its FDA approval for Parkinson’s disease and essential tremor, tFUS has seen significant advancements in clinical validation [17]. There is an increasing urgency to optimize tFUS therapeutic applications through expanding research. Recent studies have explored the impact of sonication parameters (such as intensity, duty cycle, pulse repetition frequency, and duration) on neuronal activity, revealing that these parameters can selectively promote inhibitory or excitatory effects [18,19,20]. While these findings offer valuable insights, further research is essential to fully understand and harness the therapeutic potential of tFUS.

From electrophysiological findings, intermittent tFUS was observed to briefly enhance motor excitability, whereas continuous tFUS significantly inhibited it. Our results align with those from a previous study on a large animal model, which found that tFUS at a higher duty cycle (e.g., 30%) elicited excitatory effects, whereas a lower duty cycle (e.g., 3 or 5%) led to inhibitory outcomes [21]. In this study, we analyzed MEP amplitude and observed variations resulting from the application of either intermittent or continuous tFUS. Previous research has indicated that these outcomes might be influenced by factors such as stimulus waveforms, intensity, and location, despite some conflicting findings [22,23]. While some evidence points to an inverse relationship between peak electromyography (EMG) amplitude and ultrasound intensity, Li et al. have proposed that higher ultrasound intensity might actually increase peak EMG amplitude [23]. Our study delved further into the effects of different tFUS patterns on MEP amplitude, maintaining consistent ultrasound intensity throughout. It is crucial to acknowledge that MEPs could be affected by various external factors, including electrode placement, electrode wear, and the level of signal noise, which might complicate comparisons across different animal subjects and between experimental designs. A thorough investigation into how ultrasound influences neuronal activity could significantly advance our understanding of the interplay between motor response properties and the conditions of stimulation.

Regarding the facilitation with intermittent tFUS, it was observed that the facilitation effect occurs only in the initial time interval (0–5 min post-stimulation) compared to sham stimulation. Previous studies suggest that different stimulation patterns, such as iTBS versus cTBS in rTMS, can uniquely influence neuromodulation and affect brain excitability in various ways [8]. iTBS generally increases cortical excitability, while cTBS tends to reduce it [8]. This study utilized the same number of stimulation pulses, indicating that differing after-effects might result from the patterns of stimulation (i.e., intermittent vs. continuous). In our study, we administered tFUS at very low intensities, 46.9 mW/cm² for continuous protocols and 187.7 mW/cm² for intermittent protocols, both of which are significantly below the FDA-approved safety limit of 720 mW/cm² for ultrasound intensity. To ensure equivalent acoustic energy exposure between the continuous and intermittent protocols, we adjusted the intermittent tFUS settings. This involved administering a 30 s ultrasound pulse at a 30% duty cycle, followed by a 90 s rest period, and repeating this cycle five times for a total duration of 600 s. We found that low-intensity continuous tFUS significantly inhibited motor excitability for 30 min, whereas significant facilitation was observed only within 5 min post-stimulation. The duration of changes induced by transcranial focused ultrasound (tFUS) is consistent with previous studies, which report that the facilitation of motor-evoked potentials following tFUS persists for 1 to 6 min [24]. Several parameters of the tFUS protocol might influence the duration of these after-effects, including pulse repetition rate, intensity, duty cycle, fundamental frequency, and the duration of stimulation [25,26]. Each of these parameters, alone or in combination, and especially in relation to the targeted anatomical regions, can significantly affect the impact of tFUS on brain activity. Similar findings have been observed with other non-invasive brain stimulation techniques, such as transcranial direct current stimulation (tDCS), where longer stimulation durations have been shown to result in more prolonged effects [27,28]. These observations suggest that modifying the duration of stimulation could help regulate the lasting effects of tFUS. Another factor that may influence the after-effects of tFUS is the exposure energy; higher energies are often necessary to induce neuromodulatory responses [9]. These findings highlight the importance of further research into low-intensity, safe tFUS protocols that can modulate cortical excitability and neuroplasticity. Future studies should focus on identifying optimal protocols that maximize either facilitative or inhibitive effects of tFUS.

In addition to exploring how tFUS enhances long-term potentiation (LTP)- or long-term depression (LTD)-like plasticity through electrophysiological measurements, we investigated the density of neural cells, specifically c-Fos and GAD-65, within motor cortical areas. Aligning with previous research on the excitatory and inhibitory mechanisms of brain stimulation, such as rTMS [29,30,31], our results show that intermittent tFUS upregulates c-Fos expression and downregulates GAD-65 expression. This suggests an enhancement in the synthesis of NMDA receptors. Conversely, continuous tFUS leads to an increase in GAD-65 cell numbers, implying that GABA receptors are more actively engaged during continuous tFUS application. These observations are consistent with the changes in MEPs recorded at similar time intervals. Ultrasound neuromodulation could operate through a multifaceted mechanism involving several key components. Initially demonstrated by Tufail et al., low-intensity pulsed ultrasound directly activates action potentials and synaptic transmission by stimulating voltage-gated sodium and calcium channels in the brain [5,22,32]. Further empirical evidence, as reported by Kubanek et al., shows that low-intensity focused ultrasound impacts neuronal activity and ion channel behavior in a dose-dependent manner [33]. This research also indicates that the removal of mechanosensitive ion channels, but not thermosensitive ones, nullifies the modulatory responses induced by ultrasound, underscoring the critical role of these specific channels in the process [33]. Another aspect of this modulation involves the creation of bilayer sonophores—tiny phospholipid membrane regions induced by ultrasound that lead to minor membrane deformations. These deformations produce capacitive displacement currents, contributing to short-term changes in charge dynamics. Moreover, low-intensity ultrasound is also known to affect neurotrophic factors, indicating indirect effects on neural activity and plasticity [34]. Together, these mechanisms elucidate the complex interactions between ultrasound and cellular components, providing a detailed understanding of the varied neuromodulatory effects of ultrasound.

In our rat IHC results, we found that tFUS, whether intermittent or continuous, did not cause injury to the stimulation sites at 1, 3, or 7 days post-intervention. We have developed a safe tFUS protocol that induces after-effects on motor excitability in rats. Moreover, our study confirmed that both protocols were well-tolerated. Our findings showed no significant differences in GFAP expression between sonicated and non-sonicated regions, as well as compared to the sham group, after 1, 3, and 7 days following the intervention. This was corroborated by the absence of brain damage, as indicated by GFAP expression levels. GFAP, expressed in astrocytes, serves as a reliable marker for injury response [35,36], making it a focus of extensive research in evaluating cortical lesions following brain stimulation techniques, especially tFUS [9,37]. Similarly, prior research has demonstrated that a single session of weak tFUS does not alter the typical phenotype of astrocytes within the sonicated sites [9,38]. Notably, it has also been found that tFUS applied for seven consecutive days caused no tissue damage or hemorrhage in both healthy and Parkinson’s disease (PD) mice, as evidenced by H&E and Nissl staining, thereby indicating a safe profile for the long-term application of tFUS [39,40]. Although these studies did not specifically assess microglial activation, their findings contribute to the evidence supporting the safety of long-term tFUS treatment.

The growing evidence supporting the safety and efficacy of transcranial ultrasound neuromodulation is further emphasized by its long-lasting effects post-treatment. Using functional magnetic resonance imaging (fMRI), the earlier studies have demonstrated sustained brain network plasticity, suggesting extended neuromodulatory effects on both cortical and deep brain activities [41,42]. Such lasting influence, without adverse effects, bodes well for the clinical application of ultrasound neuromodulation. Previous research, exploring safety thresholds, also highlights the reversible suppression of neural conductivity for up to 45 min following low-intensity ultrasound applications, advocating for the possibility of controlled and sustained changes in neural function [43]. The clear and enduring impact of ultrasound neuromodulation underlines its importance, positioning it as a potentially revolutionary tool in clinical settings, offering extended and focused interventions for neuroscientific pursuits.

Although intermittent tFUS demonstrated only transient facilitatory changes within 5 min, continuous tFUS delivery resulted in an inhibitory after-effect in the motor cortex lasting at least 30 min. In clinical practice, low-frequency rTMS at 1 Hz is applied to inhibit the contralesional hemisphere in stroke patients, aiding in their rehabilitation [44,45]. However, a 30 min session of 1 Hz rTMS typically induces LTD-like plasticity in the human motor cortex, which lasts for merely 10 min [46]. Therefore, a 10 min session of continuous tFUS, producing 30 min of LTD, could offer a more efficient approach than 1 Hz rTMS for post-stroke recovery treatment. Moreover, deep brain stimulation (DBS) in the subthalamic nucleus (STN) is an established treatment for patients with advanced Parkinson’s Disease (PD) [47,48]. Although the exact mechanism of DBS therapy remains partially understood, it is well-documented that most STN neurons stop firing during DBS. Our study confirms the distinct impacts of intermittent versus continuous tFUS on motor cortical excitability. These findings lead us to propose continuous tFUS as a potential therapeutic technique for PD by specifically targeting the STN. Regarding the question of whether all types of neurons respond uniformly to these treatments, we recognize the complexity inherent in neuronal responses, which vary due to differences in neuron types and circuitry within and between brain regions. The different neuronal populations exhibit varied responses to the same electrical stimulation due to differences in their morphology, physiological characteristics, and properties [49]. In our study, we specifically examined the effects of tFUS on the motor cortex. However, understanding how different neuron types, particularly in the STN, respond to continuous tFUS remains a significant challenge that requires further exploration. Hence, extending the suppressive neuromodulatory effect of continuous tFUS to deeper structures such as the STN might represent a promising non-invasive DBS alternative for treating PD in the future.

## 4. Materials and Methods

### 4.1. Animals and Preparations

Male Sprague-Dawley rats weighing between 350–400 g were sourced from BioLASCO, Taipei, Taiwan for the experiments. They were kept under a 12 h light/dark cycle at a stable temperature of 25 ± 1 °C, with unrestricted access to food and water. The experimental protocols received approval from the Institutional Animal Care and Use Committee of Chang Gung University (IACUC No. CGU107-231), adhering to the guidelines outlined in the Guide for Laboratory Animal Facilities and Care by the Council of Agriculture, Executive Yuan, ROC.

### 4.2. tFUS Procedures

**Figure 5:**
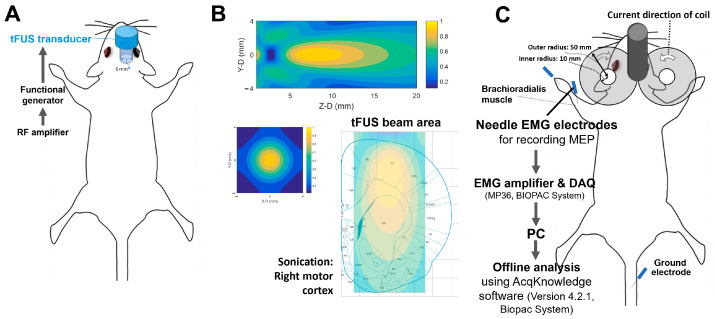
Experimental setup for measuring motor cortical excitability after transcranial focused ultrasound stimulation (tFUS) in anesthetized rats. The center of the tFUS transducer was positioned and focused on the right motor cortex (**A**). The characterizations of the tFUS pressure fields. One sagittal (Y–Z plane) and one transverse (X–Y plane) scan of ultrasound pressure distribution using a hydrophone-based US field-mapping system (**B**). The diameter and length of the half-maximum pressure amplitude of the ultrasound field and transcranial ultrasound field were within 2 and 10 mm, respectively. The placement and assembly of recording electrodes of motor-evoked potentials (MEPs) elicited by the transcranial magnetic stimulation (TMS) coil (**C**). MEP data were recorded from the brachioradialis muscle and were analyzed to evaluate any changes in cortical excitability resulting from the intervention protocols.

Before undergoing tFUS, all animals were anesthetized using a combination of Zoletil (65 mg/kg; Vibac, Carros, France) and Rompun (10 mg/kg; Bayer, Leverkusen, Germany). The rats were then positioned in a stereotaxic frame (Stoelting, Wood Dale, IL, USA) for one hour to ensure a stable baseline of neuronal activity [9,50]. A circulating water heater was employed throughout the experiment to maintain the rats’ body temperature. tFUS was administered using an ultrasound device (SonoPore KTAC-4000 NepaGene, Chiba, Japan) set to a fundamental frequency of 1 MHz. Ultrasonic gel facilitated the transmission of ultrasound through the animals’ skull, focusing on the right primary motor cortex (Figure 5A). A 6 mm diameter transducer, securely clamped to a metal stand on the stereotaxic frame, was oriented directly upwards during sonication. Acoustic pressure in the sonication area and through the rat skull was assessed using a needle-type hydrophone (HNA-0400; ONDA, Sunnyvale, CA, USA), ensuring that the pressure amplitude and acoustic deformation remained within safe limits [51]. The diameter and length of the half-maximum pressure amplitude of the ultrasound field and transcranial ultrasound field were within 2 and 10 mm, respectively (Figure 5B). Intermittent transcranial tFUS was delivered at a pressure of 0.137 MPa (accounting for transcranial attenuation) with a 30% duty cycle. The Ispta was 187.7 mW/cm². The stimulation protocol consisted of 30 s ultrasound trains followed by 90 s rest periods, repeated five times (total duration 600 s). Continuous tFUS was applied using the same pressure of 0.137 MPa at an 8% duty cycle for a continuous duration of 600 s, resulting in an Ispta of 46.9 mW/cm². This protocol was designed to investigate inhibitory effects. The acoustic exposure for both protocols complied with the US FDA’s safety threshold (Ispta ≤ 720 mW/cm²). Regarding the rationale for designing the intermittent and continuous tFUS protocols, we have added a paragraph to discuss the settings used in the current study.

### 4.3. TMS Assessment

The measurement of MEPs induced by TMS of the motor cortex is a widely used method for assessing changes in corticospinal excitability in clinical neuroscience [52,53,54]. For recording MEPs, monopolar uninsulated 27G stainless steel needle electrodes were inserted into the belly of the targeted brachioradialis muscle in the forelimb. A reference electrode was placed distally in the paw [9,50,55]. The EMG signals were amplified (gain ×1000) and filtered using a 60 Hz notch filter and 10 Hz–1 kHz bandpass filters, before being digitized at a rate of 10 kHz (MP36, BIOPAC System, CA, USA) [50].

Single-pulse TMS was directed over the motor cortex using a Rapid^2^ magnetic stimulator (Magstim, Whitland, UK) and a figure-of-eight coil (external diameter = 55 mm, internal diameter = 10 mm; Magstim) to elicit MEPs. The coil, affixed to a stereotaxic frame, targeted the left motor cortex laterally where tFUS was applied. The resting motor threshold (RMT) was determined as the minimal stimulation intensity required to produce MEPs of at least 20 μV in 5 out of 10 consecutive trials from the contralateral brachioradialis muscle [50,56]. Stimulation was then applied at 120% of the RMT at 10 s intervals. This intensity was maintained consistently throughout the experiment. MEP recordings commenced 5 min after anesthesia initiation to mitigate the effects of sedation and continued for 30 min post-tFUS to assess the effects on cortical excitability.

To reduce the frustration effects of anesthesia, MEPs were recorded 5 min following initiation of anesthesia. To examine LTP- or LTD-like plasticity induced by tFUS in the primary motor cortex, MEP amplitudes were measured starting 5 min before the tFUS procedure as a baseline and then every 5 min up to 30 min post-procedure. Thirty single test-pulse MEPs were recorded at 0.1 Hz immediately after stimulation and then every 5 min up to 30 min, using baseline intensity. For the sham group, the same tFUS protocol was followed without actual FUS application. MEP amplitude was quantified as the peak-to-peak voltage of each MEP using the AcqKnowledge software (Version 4.2.1, Biopac System Inc., Goleta, CA, USA). The amplitudes of individual MEPs were calculated and averaged for each 5 min group of EMG signals. To evaluate the impact of tFUS on cortical excitability, these data were normalized to the baseline values and expressed as a ratio change from the baseline.

### 4.4. Immunohistochemical Analysis

For IHC staining, the procedures were described in detail according to the previous study [9]. To assess the safety of tFUS protocols, we employed IHC analysis to monitor GFAP expression, a marker for astrocyte activation in response to stress or injury. Brains from rats in three different groups were collected at 1, 3, and 7 days following the completion of behavioral assessments for histological examination. The animals underwent transcardial perfusion with phosphate-buffered saline (PBS), succeeded by 4% paraformaldehyde in PBS. Subsequently, the brains were preserved in 30% sucrose at 8 °C for a week, then frozen at –80 °C before sectioning. Sections of 30 μm thickness were prepared using a Leica CM3050 S Research Cryostat (Leica Biosystems, Nussloch, Germany) and floated in PBS.

For IHC staining, we followed a previously established protocol. Sections underwent incubation in a blocking solution (10% goat serum in PBS) for 1 h at room temperature, then were treated with 0.3% hydrogen peroxide in PBS for 10 min to suppress endogenous antigen activity. Next, the sections were incubated with primary antibodies against GFAP (1:1000; AB7260; Abcam, Cambridge, UK), c-Fos (1:1000, AB11959, Millipore, St. Louis, MO, USA), and GAD-65 (1:100, AB239372, Millipore, USA) for 1 h at room temperature, followed by incubation with secondary anti-rabbit antibodies (1:200, MP-7401, Vector Labs, Newark, CA, USA) for another hour. After washing, sections were treated with 3,3′-diaminobenzidine (DAB, SK-4105, Vector Labs, USA) for 10 min for visualization. Images of the areas of interest (ROI) were captured using an Aperio CS2 digital pathology slide scanner (Leica Biosystems Inc., Buffalo Grove, IL, USA) at 40× magnification (0.25 M/pixel). Quantitative analysis of GFAP, c-Fos, and GAD-65 expression involved both manual and automated counting of the labeled cells, using images obtained from the Aperio ImageScope viewer program at high magnification. A consistent threshold for identifying specific cells within the ROI was applied across all images, which were then converted to black and white for analysis. Cell density in targeted regions was quantified using Image-Pro Version 11 (Media Cybernetics, Rockville, MD, USA).

### 4.5. Experiment Design

**Figure 6:**
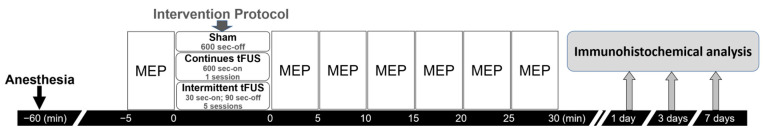
Schematic diagram of the experimental design for testing changes in cortical excitability after tFUS or sham stimulation in anesthetized rats. In this representative experiment, anesthetized rats received intervention protocols for 600 sec. Motor-evoked potentials (MEPs) were measured at baseline and 0, 10, 20, and 30 min after tFUS intervention. Subsequently, the rat brains were removed and subjected to immunohistochemistry for further investigation.

The detailed timeline of experiments is shown in Figure 6. Animals were randomly divided into three groups to examine the effects of tFUS: intermittent tFUS, continuous tFUS, and sham. All animals in these groups were subjected to their respective tFUS protocols while under anesthesia. In this specific setup, anesthetized rats underwent their intervention protocols for a duration of 600 s. MEPs were recorded both at baseline and at intervals of 0, 10, 20, and 30 min after the intervention. One, three, and five days following stimulation, the rats’ brains were extracted for immunohistochemical analysis to further explore the outcomes. To assess changes in cortical excitability, MEPs were measured both before (baseline) and after tFUS application. Furthermore, to ascertain the safety of tFUS, we evaluated the expression of GFAP in the motor cortex at 1, 3, and 7 days post-tFUS. To verify if tFUS effectively activates neurons and GABAergic synaptic terminals, as suggested by the elevated expression of the immediate early genes c-Fos and GAD-65, the rats undergoing intermittent or continuous tFUS protocols were transcardially perfused 20 min post-stimulation, and their brains were then analyzed using IHC.

### 4.6. Statistical Methods

Statistical analyses were conducted with SPSS 22 (SPSS Inc., IBM Corp., Armonk, NY, USA). The data are presented as mean values ± standard errors of the mean (SEM). We used a general linear model with a two-way repeated-measures analysis of variance (two-way ANOVA) to compare the changes in cortical excitability over time among the three groups, applying a Bonferroni correction for multiple comparisons during post-hoc tests if significant main effects were detected. Independent *t*-tests were utilized to assess differences between groups at specific time points. Additionally, we examined the temporal changes in MEPs, behavioral outcomes, and histological findings using one-way ANOVA, followed by post-hoc comparisons with Fisher’s LSD test. A *p*-value of ≤0.05 was considered statistically significant.

## 5. Conclusions

In conclusion, our study underscores the potential of tFUS as a safe neuromodulation technique that is capable of inducing neural plasticity and activating neural cells. The applicability of our findings to human and disease models warrants further investigation in future studies.

## Data Availability

The raw data supporting the conclusion of this study are available from the corresponding authors on reasonable request.
